# Reliability and structural validity of the Danish Short 4-item version of the Center for Epidemiological Studies Depression Scale for Children (CES-DC4) in adolescents

**DOI:** 10.1186/s12887-022-03451-7

**Published:** 2022-07-02

**Authors:** Christine Leonhard Birk Sørensen, Therese Koops Grønborg, Karin Biering

**Affiliations:** 1 Department of Occupational Medicine, University Research Clinic, Danish Ramazzini Centre, Goedstrup Hospital, Hospitalsparken 15, 7400 Herning, Denmark; 2 Department of Public Health, Aarhus University, Bartholins Allé 2, 8000 Aarhus C, Denmark; 3 Department of Clinical Epidemiology, Aarhus University Hospital, Olof Palmes Allé 43-45, 8200 Aarhus N, Denmark

**Keywords:** Psychometric properties, Depression, Adolescents, Reliability, Questionnaires, Self-report

## Abstract

**Background:**

The 4-item version of the Center of Epidemiological Studies Depression Scale (CES-DC_4_) is a self-reported questionnaire used to measure depressive symptoms in adolescents, but the psychometric properties of the scale have been tested to only a limited extent. The aim of this study was to examine the reliability and structural validity of the Danish CES-DC_4_ in 9^th^ graders.

**Methods:**

Using a sample of 72 adolescents 15 to 17 years of age from five 9^th^ grade classes, the reliability of the CES-DC_4_ was determined by a test–retest study at a 2-week interval. Descriptive statistics of the adolescents were presented, and internal consistency, structural validity, reliability, and agreement between tests were evaluated. The structural validity of the scale was tested by confirmatory factor analysis (CFA), and the sumscores of the test and retest were presented.

**Results:**

The estimated Cronbach’s α was 0.61 (95% CI 0.50; 0.71). Inter-item and item-rest correlations indicated that one of the four items (item 20) did not fit well on the scale. CFA found a one-factor model suited for the scale, but the factor loadings indicated that item 20 contributed the least to measure the factor (0.29). Sum scores ranged from 0–9 within a possible interval of 0–12. There were no signs of systematic error of the scale. Limits of Agreement (-3.01; 3.79) were broad. The standard error of measurement (SEM = 1.25 point (95% CI.1.05; 1.47)) and intraclass correlation (ICC(2,1) = 0.60 (95% CI: 0.44; 0.73)) calculations showed low reliability of the CES-DC_4_.

**Conclusion:**

This study found low reliability of the CES-DC_4_ with low estimates of ICC and Cronbach’s α. The CES-DC_4_ needs revision, and removal of item 20 and adding more items from the CES-DC should be considered.

**Supplementary Information:**

The online version contains supplementary material available at 10.1186/s12887-022-03451-7.

## Background

Depression is the largest contributor to years lived with disability, and the World Health Organization (WHO) estimates depression affects 400 million persons worldwide [1]. Depression in adolescents can result in severe impairments that often continue into adult life [2]. In Denmark, there has been a serious negative development in depression among adolescents. The incidence rate (IR) of recurrent depression among 15- to 20-year-olds has increased from 0.12 (95% CI: 0.12;0.12) in 1970–1984 to 5.95 (95% CI: 9.94;5.95) in 2005–2016 [3]. Moreover, a nationwide survey has shown a growing number of adolescents who rate their mental health as poor. Recent results from 2021 showed a record high prevalence of adolescents with poor mental health with a percentage of 21.2% among 16- to 24-year-old men and 34.4% among 16- to 24-year-old women [4]. Therefore, early identification of depressive symptoms is of great importance. However, a reliable and valid measurement instrument is needed. Self-report questionnaires have been found suited for this aim because of their easy, low-cost, and rapid administration [5].

The Center for Epidemiological Studies Depression Scale for Children (CES-DC) is a widely used measurement in North America [6, 7], Central America [8], Europe [9–14], Asia [15, 16] and Africa [17], where the psychometric properties of the scale have been evaluated in order to use the scale as a screening tool. The CES-DC is developed to assess depressive symptoms in children and adolescents aged 6- to 17-years in the general population, while other scales to detect depressive symptoms in childhood and adolescents, e.g. the Children's Depressive Inventory (CDI), have been developed for clinical populations [18]. The CES-DC was derived from the adult version (CES-D) and consists of 20 items [7].

Studies of the CES-DC have generally shown excellent internal consistency (Cronbach’s α = 0,82–0.91) [6, 9, 13, 15–17, 19], apart from one study, that found acceptable internal consistency (Cronbach’s α = 0.67) [10]. Two studies reported excellent test–retest reliability (ICC = 0.71–0.85) [16, 17], while another study found acceptable test–retest reliability among adolescents, but not among children [19]. Concerning the structural validity of the scale, the results of the inter-item correlations have been inconsistent [9, 16]. Several studies found a 4-factor model suitable for the scale [9, 10, 12, 13, 15, 16, 20]. Assessing the convergent validity, by studying correlations with other scales that measure similar constructs, has supported the conclusion of good construct validity. A good construct validity of the tests is supported by correlation with other scales that measure similar constructs [9, 13, 15–17, 20]. As a screening tool, the CES-DC has showed good sensitivity (80.0%-81.9%), acceptable to good specificity (57.0%-71.9%) [9, 14, 19], poor positive predictive value (13%), and good negative predictive value (97%) compared with diagnosis. The scale has shown good criterion validity among adolescents but not among children [19].

In 1990, Fendrich et. al. developed a short 4-item version (CES-DC_4_) of the CES-DC to make screening of depression less time-consuming. Items that had the highest loadings in each of the four factors were selected for the short scale (items 12, 15, 18, and 20). CES-DC_4_ showed a sensitivity of 62% and a specificity of 61%. Moreover, the CES-DC_4_ showed acceptable internal consistency (Cronbach’s α = 0.64) [19]. Houghton et al. studied the psychometric properties of the CES-DC_4_ in adolescents in Ireland. Correlations with the Children’s Depression Inventory (CDI) (*r* = 0.497) and the self-esteem scale of the Child Health Questionnaire (CHQ-CF87) (*r* = -0.54) proved acceptable convergent validity, but they found poor internal consistency (Cronbach’s α = 0.583). Houghton et al. argued that the short scale has great potential as a screening tool because there were no problems during the administration of the CES-DC_4_ [11]. Other scales to measure depression symptoms have shown problems during administration, e.g. there were a high number of non-respondents to the "feel like crying" question in the CDI, and Hougthon et al. found that the "feel like crying" question was significantly less likely to be completed and boys responded significantly less than girls [21].

The CES-DC_4_ has been translated and adapted into Danish (not published) and used in large cohort studies of Danish adolescents since 2004 [22, 23]. The scale is preferable to use in large or longitudinal cohort studies because it is short, and a version for both children and for adults is available. However, neither the reliability nor the validity of the Danish version of the CES-DC_4_ has been examined. The primary aim of this study was to test the reliability of the CES-DC_4_ in Danish 15- to 17-year-old adolescents. The secondary aim of this study is to test the structural validity of the CES-DC_4_ in the same population.

## Methods

### Participants

For this study, 71 public and private schools with regular 9th grades in the geographical area of Aarhus, Favrskov, Silkeborg, and Skanderborg municipalities were invited to participate through email from the corresponding author. The geographical area was chosen to represent both larger cities and small towns. Schools for adolescents with special needs were not invited. Inclusion criteria were adolescents above 15 years of age with the ability to read and understand Danish. The aim was to include a sufficient number of adolescents to fulfill COSMIN’s criteria for an adequate sample size of at least 50 participants to evaluate reliability, taking risk of dropout in the retest into consideration [24, 25]. Of the 71 invited schools, 10 schools declined to participate, 58 did not react to the request and three schools agreed to participate, resulting in participation of five 9th grade classes.

### Variables

**Fig. 1:**
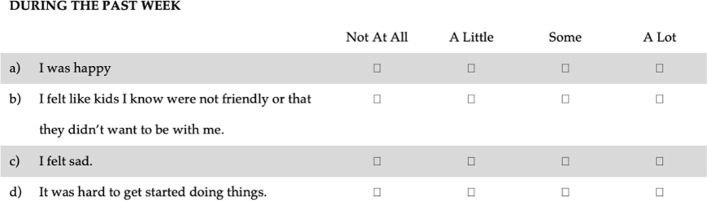
The original English version of CES-DC_4_

**Fig. 2:**
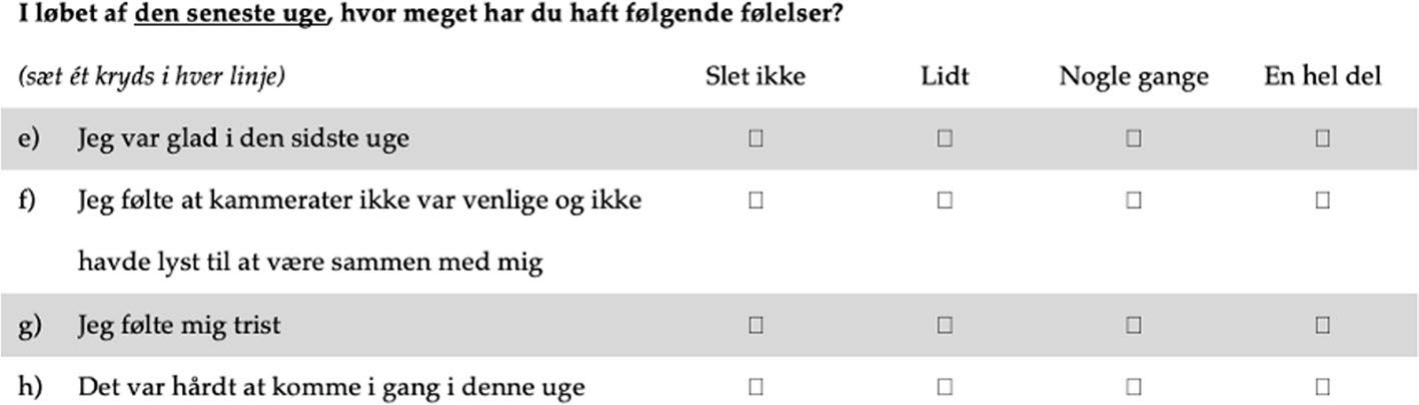
The Danish version of CES-DC_4_

Data were collected through a printed questionnaire. The questionnaire contained questions about age, sex, and the CES-DC_4_. The original English version of the CES-DC_4_ is shown in Fig. 1 and the Danish version is shown in Fig. 2.

The CES-DC_4_ measures depressive symptoms and consists of four items scored from 0–3 on a 4-point Likert scale. The first item is positively phrased and is reversely scored. The sum scores of the scale ranges from 0–12. The higher the score, the more depressive symptoms.

### Study design

In the COSMIN guidelines, reliability as a measurement property is defined as “the proportion of the total variance in the measurements, which is because of ‘true’ differences between patients” [26]. In this study, the test–retest reliability was evaluated, with a 2-week interval between test and retest. The time interval was chosen to prevent recall from test to retest, and the adolescents’ mental health was expected to be stable during this period [27]. The structural validity of the scale was tested in the adolescents who completed the test questionnaire. The hypothesis of the study was that a one-factor model was suitable for the scale.

### Procedure and location

Data were collected from January to March 2020. The test and retest both took place as self-administered questionnaires in the adolescents’ classrooms while they were sitting at their seats. Before the test, there was an introduction including a presentation of the researcher, the aim of the research, information about voluntary participation and anonymity, and the practical execution of filling out the questionnaires. The adolescents were told to leave the questionnaire unanswered if they did not wish to participate. Every student got an individual emoji on their questionnaire together with a printed emoji they could keep in their phone cover or pencil case, which they had to remember until the retest. At the retest, they got a questionnaire with the same emoji. In that way, the test and retest could be paired, and the adolescents kept their anonymity since the authors never were able to identify which questionnaire was answered by whom. An emoji was chosen because the authors expected the adolescents would be able to remember an emoji better than a specific number. There were no incidences of students not remembering their emoji at the retest. The introduction and information regarding the practical execution were the same at the test and the retest, and the same emoji was used at both test and retest. At retest, the adolescents did not know the sum score of their initial test questionnaire.

### Statistical analyses

Descriptive statistics were calculated for the age and sex data used in the structural validity analyses and the reliability analyses. and the time interval between test and retest was calculated in the sample used in the reliability analyses. Age and sex were presented with the number, percentage, maximum value, and minimum value. The time intervals were presented with mean, standard deviation (SD), maximum value, and minimum value. A dropout analysis of the descriptive data was performed in the adolescents who answered the first questionnaire and those who answered both questionnaires (*n* = 29).

To analyze the internal consistency of the CES-DC_4,_ Cronbach’s α and the Omega coefficient were estimated, and item-rest correlations and inter-item correlation were evaluated [27, 28].

In COSMIN guidelines, structural validity is defined as an aspect of the measurement property construct validity as “The degree to which the scores of an instrument are an adequate reflection of the dimensionality of the construct to be measured” [23]. The CES-DC_4_ follows a reflective model, the scale measuring symptoms that are consequences of a disease. The dimensionality of the CES-DS_4_ was evaluated using the first round of test scores (*n* = 95) by confirmatory factor analysis (CFA), where items were analyzed as categorical measures with a weighted least-squares means and variance adjusted(WLSMV) estimator. A one-factor model was evaluated by several goodness-of-fit and badness-of-fit indices. The Comparative Fit Index (CFI) assesses fit relative to a null model, and the Tucker-Lewis Index (TLI) adjusts for the number of model parameters. CFI ranges from 0 to 1, and TLI also ranges from 0 to 1, occasionally presenting values a little below 0 and a little above 1. For both indices values above 0.9 indicate acceptable fit. The root mean square error of approximation (RMSEA) expresses the lack of fit per degree of freedom in the model, and the standardized root mean square residual (SRMR) is the average of the differences between the observed and predicted correlations, values below 0.08 indicating a good fit in both [27]. The sum score for the two samples used in the structural validity analyses and the reliability analyses were presented with mean, SD, maximum value, and minimum value. To obtain estimates of the confidence intervals of Cronbach's alpha, Omega coefficient, inter-item correlations, item-rest correlations and SEM, we used bootstrap methods.

To evaluate the test–retest reliability the random error, systematic error, agreement between tests, and association between tests were assessed. Limits of agreement (LoA) were estimated to give an indication of the size of the random error. To evaluate the systematic error, the mean difference between the two test scores was estimated. A Bland–Altman plot of differences between the sum scores at test and retest against the means of the sum scores was generated to assess the extent of agreement between the tests and the systematic error. The correlation between the two test scores was estimated to assess the strength of the association between the test and retest. The variances between test and retest, between the adolescents, and within each adolescent were estimated. To evaluate the reliability of the CES-DC_4,_ the standard error of measurement (SEM), and the intraclass correlation (ICC (2, 1) were estimated. The ICC[2, 1] was estimated by a two-way random effect model for single measurement to assess the absolute agreement, and the estimation of SEM was based on the same model [27, 28].

The statistical analyses were performed with Stata16 software [29], except for the CFA, which was performed in R version 1.2.5019 [30] and the R package lavaan [31].

## Results

### Participants

**Fig. 3:**
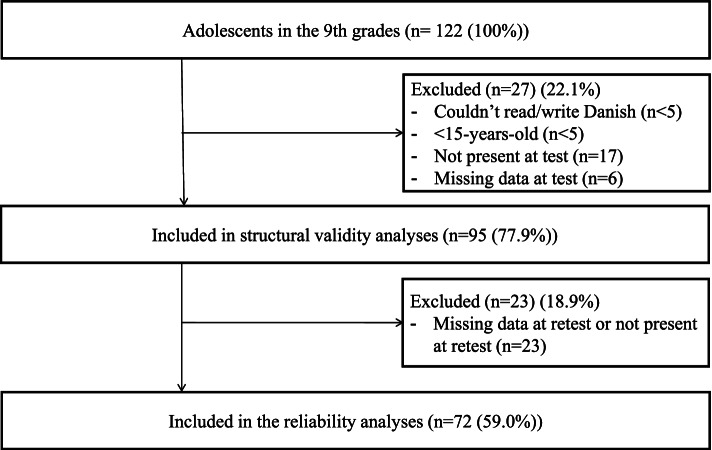
Flowchart of participants

The five 9th grades consisted of 122 adolescents, 72 (59%) of whom were included in reliability analyses and 95 (78%) in the structural validity analyses. Adolescents were excluded from the reliability analyses if items were missing or if the adolescents did not attend both test and retest, and excluded from the structural validity analyses only if items were missing at the test (*n* = 6). Items were categorized as missing if there was no indication or a double indication (Fig. 3). Thus, the sample size fulfilled the recommendation of providing at least 50 participants in a reliability analysis and at least six times the number of participants per items but less than100 participants in the evaluation of the structural validity [24, 25].

Table 1 presents the age, sex, and mean time interval between test and retest for the included adolescents in the two samples used in the structural validity analyses and in the reliability analyses.

**Table 1:** Characteristics of the study population

	Structural validity analyses (*n* = 95)	Reliability analyses (*n* = 72)
Age, 15/16–17 (%)	79/16 (83.16/16.84)	60/12 (83.33/16.67)
Sex, m/f (%)	52/42 (55.32/44.68)	39/33 (54.17/45.83)
Mean time interval between tests (SD) [range]	N/A	14.72 days (1.56) [13.96–18.02]

### Response analysis

The dropout analysis showed no significant differences between excluded and included adolescents for the reliability analyses concerning age, sex, and the scores for items 12, 15, 18, and 20 at both test and retest (data not shown).

### Internal consistency

The Cronbach’s α was 0.606 (95% CI: 0.503; 0.709), which is below the recommended interval of 0.7–0.9 [27], and the estimated Omega coefficient was 0.661 (95% CI: 0.583; 0.738). The inter-item correlation ranged from 0.102 to 0.492 (see Table 2). The low correlations between items 12 and 20 (*r* = 0.183 (95%CI: 0.019; 0.331)) and items 15 and 20 (*r* = 0.102 (95%CI: -0.060; 0.261)) were lower than the recommended limit of 0.2 and did not support the conclusion of a unidimensional scale. The item-rest correlation ranged from 0.218 (95% CI: 0.054; 0.381) to 0.560 (95% CI: 0.439; 0.680), with the lowest correlation for item 20 and the highest correlation for item 18. Furthermore, the analyses showed that the Cronbach’s α would be higher (α = 0.678) if item 20 was deleted from the scale.

**Table 2:** Inter-item correlations in CES-DC_4_

	**Spearman’s ρ (95% CI)**			
	Item 12	Item 15	Item 18	Item 20
Item 12	1.00			
Item 15	0.304 (0.127; 0.467):	1.00		
Item 18	0.492 (0.358; 0.617)	0.436 (0.291; 0.555)	1.000	
Item 20	0.183 (0.019; 0.331)	0.102 (-0.060; 0.261)	0.208 (0.040; 0.356)	1.000

### Structural Validity

The results of the CFA with one factor showed good fit with a CFI of 1.000, a TLI of 1.038, a RMSEA of 0.000 (95% CI: 0.000, 0.175), and a SRMR of 0.036. A one-factor model being found suitable for the scale, the mean sum score at test and retest was calculated (presented in Table 3).

**Table 3:** Sum scores of the samples

	Structural validity analyses (*n* = 95)	Reliability analyses (*n* = 72)
Mean sumscores test (SD) [range] Mean sumscores retest (SD) [range]	3.07 (1.99) [0–9] N/A	3.14 (1.92) [0–9] 2.75 (2.01) [0–7]

**Fig. 4:**
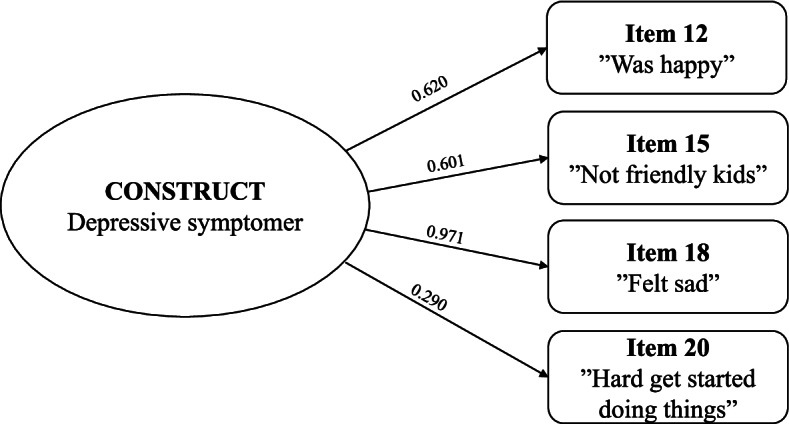
Factor structure and factor loadings of the CES-DC_4_

The factor loadings showed the highest loading for item 18 (0.971) and the lowest loading for item 20 (0.290), indicating that item 18 contributed the most to the factor and item 20 contributed the least. Factor loadings and factor structure are presented in Fig. 4.

### Reliability

The estimated ICC (2,1) was 0.604 (95% CI: 0.435; 0.732) and the SEM was 1.247 (95% CI: 1.053; 1.466).

### Agreement

**Fig. 5:**
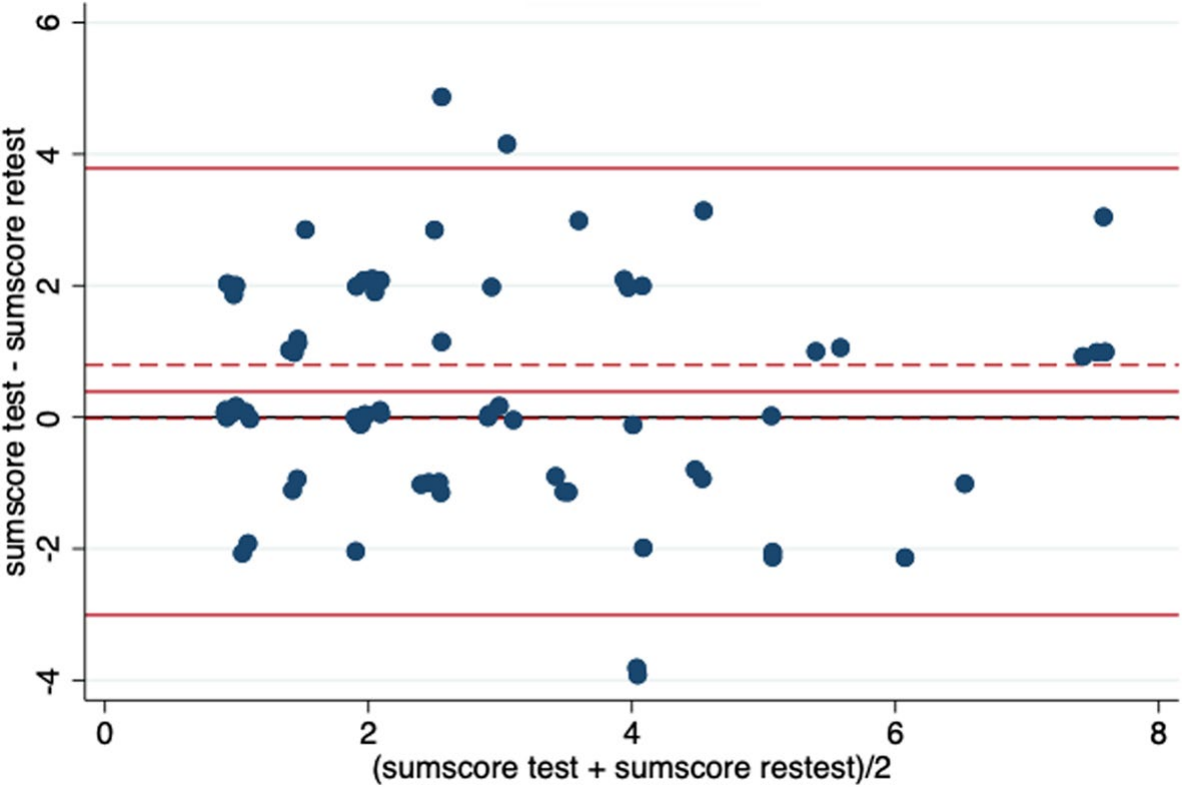
A Bland–Altman plot of differences between test and retest against the means

There was no statistically significant difference between test and retest (mean = 0.389 (95% CI: -0.018; 0.796)). The estimated LoA were -3.007 (95% CI: -3.694; -2.320) to 3.785 (95% CI: 3.097; 4.472), which is visualized in the Bland–Altman plot in Fig. 5. The estimated correlation between test and retest was 0.613 (95% CI: 0.445; 0.740).

## Discussion

The present study is the first to assess the reliability and structural validity of the Danish CES-DC_4_ in adolescents. Only two other studies have assessed the psychometric properties in the English CES-DC_4_ in other contexts in terms of internal consistency [11, 19] and convergent validity [11]. We found low internal consistency, with a low Cronbach's α and inter-item correlations that did not support a unidimensional scale. However, the CFA supported a one-factor solution for the scale. We found low reliability of the scale, but the agreement between the test and retest did not show signs of systematic error.

### Measurement property evidence

The measurement properties of internal consistency were not sufficient. The estimated Cronbach’s α (0.606 (95% CI: 0.503; 0.709)) was below the recommended limit of 0.7 [27]. Other studies on the short scale found a Cronbach’s α on 0.583 and0.640 [11, 19], which were not statistically different from the Cronbach's α of this study (*p* = 0.784) [32]. Studies examining the full scale primarily found excellent internal consistency (Cronbach’s α = 0.82–0.91) [6, 9, 13, 15, 17, 19], while one study found acceptable internal consistency (Cronbach’s α = 0.67) [10]. Moreover, the inter-item and item-rest indicated problems with item 20. The inter-item correlations between item 12 and item 20 and between item 15 and item 20 were below the recommended limit of 0.2 and did not support the conclusion of a unidimensional scale [27]. The low item-rest correlation for item 20 (*ρ* = 0.218) indicated that item 20 did not contribute much to the distinction between adolescents with low and high scores on the rest of the items. Furthermore, deleting item 20 would improve the internal consistency, indicating that item 20, whether it was hard to start doing things this week, could be measuring a different dimension. Two other studies reported inter-item correlations of the full scale and found conflicting results. In one study, the correlations ranged from 0.39 to 0.57, and no problems with item 20 were identified [16], while the inter-item correlations ranged from 0.07 to 0.77 in another study, with a total of 28 very low correlations (< 0.2) [9].

The one factor model showed a good fit within the recommended levels of CFI ≥ 0.95, TLI ≥ 0.95, RMSEA ≤ 0.06, and SRMR ≤ 0.08 [27]. Even though the inter-item correlations did not support the conclusion of a unidimensional scale, the CFA supported a one-factor solution of the CES-DC_4_. However, as the internal consistency analysis showed, problems with item 20 were also present in regard to the factor loadings, which showed that item 20 contributed the least to the factor.

The measurement properties of the reliability were not sufficient because the ICC ^(2,1)^ (0.604 (95% CI: 0.435; 0.732)) was below the recommended limit of 0.7 for measurements of groups. The value of reliability parameters like Cronbach's α and ICC depend on the heterogeneity of the study population and number of items with higher reliability estimates as heterogeneity and number of items increase. The homogeneity of our study population along with the small number of items may contribute to the low estimates of reliability in our study. Moreover, when studying the reliability in a test–retest study, the construct of interest is assumed to be stable in the chosen time interval. If the depressive symptoms in the adolescents were not stable during the 2-week interval, this will cause bias in the reliability estimates [27].

The absence of any signs of systematic error suggests that the measurement properties of the agreement were sufficient. However, the estimated LoA (-3.007; 3.785) were broad considering the sum scores in this study ranged from 0–9. Other studies examining the full scale found both a higher (r = 0.85) and a lower correlation (r = 0.51) between test and retest than the one estimated in this study (r = 0.613 (95% CI: 0.445; 0.740)) [4, 15]. Therefore, the result of the broad LoA is not surprising.

### Practical relevance

The estimated SEM (1.247 (95% CI: 1.053; 1.466)) was high considering the range of the sum score. Measurement error can have a profound effect on the SEM. A small change in the sum score could reflect the measurement error rather than a change in the construct of the adolescent when changes are measured over time.

### Strengths and limitations

The study has several strengths concerning internal validity. The risk of information bias is considered to be minimal. Mis-classification was not suspected because the adolescents were anonymous and had the possibility to answer their questionnaires privately. Unfortunately, only 59% of the total sample were included in the reliability study. However, a comparison of excluded adolescents who only attended the test and adolescents who attended test and retest showed no significant difference between the groups in terms of age, sex and item scores. Therefore, the excluded adolescents were likely missing at random (data not shown). Nobody declined to participate, and most of those excluded were not present at either the test (13.9%) or retest (18%). The missing answers were not related to a specific item. If we had used electronic questionnaires, the number of missing items might have been lower. However, the number of persons with missing items was relatively low (7 persons (5.7%)), thus we do not expect that this would have affected the results. Therefore, the risk of selection bias is considered to be minimal.

Another strength of the study is that the adolescents answered the questionnaire in the exact same setting each time.

### Generalizability

The study sample consisted of regular 9th graders, while adolescents with special needs, dyslexia, or lack of Danish language skills were excluded. Only 3 of the 71 invited schools participated. Whether the schools wished to participate or not is not expected to cause selection bias, because it is not expected to be related to the mental health of the adolescents. Moreover, among the included schools were both schools in a large city (> 350,000 inhabitants) and one in a small town (< 7000 inhabitants). Therefore, the results are expected to be generalizable to regular 9th graders in the rest of Denmark.

### Instrument changes

To our knowledge, no studies have estimated the ICC of the CES-DC_4_, whereas studies examining the full scale found a high ICC (0.71–0.82) and thereby good reliability of the full scale [16, 17]. Four items may not be enough to measure depressive symptoms in adolescence as the mood of adolescents varies considerably [33]. Adding more items to the short scale should therefore be considered.

Moreover, choosing the item with highest factors loading from each of four different dimensions of the full scale may not have been the most optimal method to develop the short scale, which is used as a unidimensional scale. Other approaches should be considered to ensure the unidimensionality of the short scale.

### Future Research

The validity of the Danish CES-DC_4_ still needs further evaluation to determine the psychometric properties in a Danish context. The content validity of the scale is especially relevant to assess as other researchers have suggested that the scale measures emotional tumult in adolescents rather than depression symptoms [9].

A possible explanation for the low reliability estimates in this study is that the chosen time interval was too long for the construct to remain stable. Further studies should include investigation of whether a shorter time interval would be more suitable for a test–retest study of the scale. A gold standard to measure depressive symptoms, such as a diagnostic interview, at the same time as the test and retest would make it possible to detect changes in the construct.

This study found problems with item 20 regarding the internal consistency. The problems with item 20 could be due to the CES-DC_4_ being developed for children, not adolescents. A cultural adaption of the questions for adolescents should be considered as item 20 ("It was hard starting doing things") may have different meanings for children and adolescents and does not necessarily reflect depressive symptoms in adolescents.

Moreover, responsiveness should be examined before using the scale to track changes over time since the rather large SEM could indicate that the responsiveness of the scale is questionable.

## Conclusion

This study found low reliability of the CES-DC_4,_ with low estimates for ICC and Cronbach's α and a high SEM. However, the results are comparable with the few other studies examining the short scale. Concerning the structural validation, a one-factor model was found suitable for the scale. The inter-item correlations, item-rest correlations, and factor loadings indicated problems with item 20.

At the moment, a better alternative to measure depressive symptoms in adolescents in a short, quick way, has not been identified. The authors suggest that the choice of items for the short scale is reconsidered to secure a better reliability in adolescents.

## Supplementary Information

**Additional file 1.**

## Data Availability

The dataset analyzed during the current study are available in the supplementary file.

## Abbreviations

CDI-S: Children’s Depression Inventory
CES-DC: The Center for Epidemiological Studies Depression Scale for Children
CES-D: The Center for Epidemiological Studies Depression Scale
CES-DC_4_: The Center for Epidemiological Studies Depression Scale for Children 4 item version
CHQ-CF87: The Child Health Questionnaire
dif.: Difference
ICC: Intraclass correlation
LoA: Limits of Agreement
SD: Standard deviation
SEM: Standard error of measurement
WHO: World Health Organization
